# Coccobacteria Septica

**Published:** 1879-05

**Authors:** 


### ARTICLE XXIV.

COCCOBACTERIA SEPTICA. OBSERVATIONS MADE BY DRS. TH. BILLROTH AND F. EHRLICH. (*Archiv. für Klinische Chirurgie.* Vol 20th, part 2d.) Translated from the German by J. W. DAL, M. D.

*Observations concerning the occurrence, further development, and course of coccos vegetations in completely closed abscesses.*

I have for more than a decade, advanced the opinion, and endeavored to confirm the same, by my clinical experience, that the wounded always infect themselves from their wounds, resulting either in febrile or pyemic action, as the case may be, and that therefore, above all things, wounds must be treated very carefully, especially protected from irritation and infection. It is to be hoped that from the following observations, I shall not be accused of underestimating the dangerous causes which may act upon wounds from without. I have also in my work upon coccobacteria, shown clearly enough, that these little vegetations can carry within or affix to themselves, inflammatory or any other variety of infectious ferments, and because of their ubiquity, and especially their rapid propagation, in badly ventilated rooms provided for the wounded, become the most dangerous carriers of those ferments. Frisch* has proven through his extensive investigations, that the taking of inflammatory and infectious matter from decaying fluids and inflammatory products, by inoculation, was induced solely through the vegetation of cocci, because the inoculation of inflammatory products and decaying fluids

* Experimental studies on the spreading of decaying organisms in the tissues, etc., Dr. Frisch, Erlangen, 1874.

free from cocci produced no action upon the cornea. With reference to practical surgery, these observations are quite sufficient to make it an imperative duty to secure the utmost cleanliness in the bandages and sick wards, as for practical purposes it is of little importance, whether the infectious material is inseparably adherent to these algae or is produced during their vegetation.

Many interesting problems remain to be solved in this direction, in the interests of science. Frisch has shown that coccos can vegetate a certain time (forming a star shaped figure), without causing inflammation, the cocci disappear, or fall out, the slight defect being replaced in a remarkably short time, without being accompanied by keratitis ; from this I draw the following conclusions : among other things it is evident, that it is not the chemical or vital act itself, which causes the inflammation or phlogistic zymosis.

I arrived — basing my opinion upon purely morphological grounds—at the conclusion, that all coccobacteria vegetations which to us appear alike, do really belong to the same species of plants, that the difference of their action would depend on whether they had previously vegetated in nutritive fluids, which contained septic or phlogistic ferments or in innoxious or indifferent fluids. Different generations of coccobacteria vegetations would, according to this view, be at times entirely harmless, at other times excessively virulent, provided their germs be developed in a tissue. As an illustration, I will mention the following experiment: the eye of a rabbit will suppurate under all conditions, if a thread be drawn through the same, and permitted to remain. In the pus, coccos vegetations will always be established.

No one will maintain that it is not the thread, but the coccobacteria vegetations which, in this instance, is the causative element in the suppuration of this bulb. Inoculation, with this pus upon the healthy cornea of a rabbit, causes, by means of these coccos vegetations, intense keratitis and conjunctivitis ; perhaps, also, panophthalmia, followed by phthisis bulbi. From this I conclude that every coccos vegetation found in decomposing pus assumes infectious properties. In other words, that coccos vegetations only then become infectious when they themselves have been infected. It would be an important argument favorable to this view were it possible to disinfect coccos vegetations that have proven their infectious qualities without thereby disturbing their vegetative power, and then again to render them infectious by carrying to them certain kinds of matter. It is to be regretted that our methods of investigation have not yet advanced far enough; it can, therefore, not be denied for the present, that we are entitled to assume different species, according to the chemical action which takes place during the presence of vegetating coccobacteria in tissue fluids and secretions. It must also be admitted that on account of the small size of the object, we are not able, even with our best instruments, to notice any morphological differences between them.

Whether there do exist coccobacteria, which are pathogenic and remain pathogenic, or whether the different species of coccobacteria under certain circumstances are more or less alike, and then in turn become indifferent (perhaps simulating the mucorin spores, which at times become active ferments, while spores of the same vegetation may develop into ordinary mold, but these latter spores may in turn again become a ferment) this it is at present impossible to decide. We can only declare ourselves in favor of one or the other theory, on the ground of possible probabilities and analogy; or we must carry our observations into the fields of purely theoretical medicine or metaphysics, believing either the one or the other.

As certain as it appears to me from the investigations before me, that the taking of infectious materials in wounds from the outside, is mainly transmitted through these coccos vegetations, so important it appears to me to admit without farther comment, that those tissue changes which occur (according to the vulgar idea, decomposition with formation of odorous products) during acute inflammation and decay, are only brought about by the vegetative action of coccos, or through a ferment which they alone can transmit.

Even though we have, through the terms "odorless," "smelling," "foul smelling secretions," etc., provided for our perception, yet it must be evident that all these terms tell us nothing about the composition of these secretions, whether they be phlogistic, pyogenous or other varieties of ferments or fermentation products. It has been sufficiently demonstrated that the small quantities of strongly odorous bodies as sulphuretted hydrogen, butyric acid, ammonia, etc., which are found in the secretions, though they certainly are not entirely harmless to the tissues and blood, neither alone nor in combination possess the terribly poisonous action which appears to be fermentative and which at times is developed by these secretions.

It may be looked upon as pretty well established that the phlogistic and putrid ferment and poison is an odorless body; hence, we draw the conclusion that because its development is often accompanied by the production of odorous bodies, that the first are there also where the latter appear, without being able to exclude their existence, where our sense of smell can detect nothing. Paradoxical as this may seem to the laity, it would be wholly unwarrantable, according to past experiences, to assert that it is possible to diagnosticate the presence of pathogenic or septogenic ferments by the sense of smell.

If it were possible to smell the poison of typhus, intermittent fevers and cholera, we should indeed give them a wide berth. The ferments in question are probably all odorless; whether the products of their action smell, is entirely a secondary question, according to a scientific view, and it depends on numerous circumstances sometimes entirely irrelevant, such as for instance the formation of the *fleur de vin*, which takes place entirely independent of the formation of alcohol and the process of fermentation.

When we designate the history of a wound as aseptic, we mean thereby that the secretions of the wound are odorless. Most wounds show no sign of inflammation during this course, and there are but few, if any, coccobacteria found in the secretions. The wounded have no fever, or at least it is very slight; and yet a wound may heal without inflammation or odorous secretions, but it may contain many coccobacteria, with a high degree of fever.

It follows, therefore, that coccobacteria vegetations, odorous secretions and fever do not always occur at the same time, viz., that one is not directly dependent upon the other.

Inflammatory and traumatic fevers arise, according to my hypothesis, from the resorption of degenerating or decaying products from the inflamed and injured tissues, and through their direct or indirect action upon the blood and nervous system; whether or not these decaying products smell, can prove nothing for or against my views. A long time ago I called attention to the fact, that if wounds be treated freely exposed, the secretious will mostly be odorless. According to the popular acceptation, this is the aseptic method par excellence. That the same results are obtained by Lister's method, that the same in truth may be utilized in a much greater degree than at present, I regard as an established fact. Notwithstanding this, there is an error somewhere in the physio-pathological establishment of Lister's method of treating wounds, which, however, cannot be looked upon as a reproof.

It has happened not only to the old alchemists, but to many of our most accurate modern observers, that during their investigations they have found something entirely different, perhaps indeed practically better than that which they theoretically sought for. The throwing together of botanical, chemical and clinical investigations in such a manner that an accidental discovery shall at once explain or refute everything in all directions, has brought about a state of unpleasant restlessness and bittter animosity in the discussion of the observations themselves, to establish and understand the connection of which must be our constant aim, without thereby laying aside our sensible scientific resignation to live for the present with questions which we can but try to solve, to work, to teach, and treat our wounded. As upon other domains of science we can only conquer step by step the domain of knowledge, and nobody is therefore free from error.

I have in these preliminary remarks defined my position with reference to the bacteria, septic, and Lister questions, which has not been altered by any of the late investigations, although much of that which I formerly observed and communicated, may be otherwise construed, and many of my hypotheses fall or must be modified. The following investigations furnish, probably, verifying rather than new facts; yet, they will serve to show my continued interest in the most important question of the day. There are certain parts of these researches which imperatively demand clinical material, and to these I wish to add a few contributions although they raise more new issues than they can solve.

It has always appeared to me of paramount importance to know whether coccobacteria vegetations occur in completely closed abcesses. If this be the case, it appears to me proven (excluding the *generatio æquivoca*) that through the respiratory organs, or the alimentary canal, germs enter into the blood, that there they retain their vitality for a certain time at least, and only require to be brought under circumstances favoring their development, in order to vegetate. That this vegetation is feeble and not of long duration, is to be expected *a priori*, because plants as a rule only employ gas for their nourishment, which exists free or absorbed in fluids, they not being able to decompose organic and inorganic compounds such as water; and furthermore, plants as well as animals, perish in their excretion products.

It follows, therefore, that the coccobacteria vegetations cannot be expected to develop luxuriantly if their excretion products cannot be removed. It is usually assumed that they chiefly consume oxygen, but this is by no means certain; perhaps they chiefly require carbonic acid gas, if they are to flourish luxuriantly, certain it is that they demand, besides the introgenous compounds or carbonic hydrates, certain other component parts of the air.

We will, for convenience sake, divide the cases in which coccobacteria occur within closed abcesses, into primary and secondary or metastatic abcesses.

I. *Cases of coccobacteria vegetations in primary subcutaneous inflammatory abcesses.*—In my former work (Coccobacteria, p. 89) I cited two cases: Micro-coccos chains in a suppurating blood extravasation on the lower third of the thigh; and in an abcess which had formed about an anchylosed elbow joint in which forced action had been employed to render it movable.

I can add to these, two more very similar ones, with widely varying results.

Anna Schlosser, æt. 59. After she had scrubbed floors for several days, an acute præpatellar abscess formed on the left knee, and for this she was brought to the hospital, on the 10th day after the beginning of the inflammation. The abscess was punctured, giving exit to odorless, slimy, viscid pus, which contained moderate quantities of plainly marked though latent micro-coccos chains.

Barbara Dolcher, æt. 23, a strong, well developed servant girl, had, on the 3rd of December, 1875, scrubbed the floor with much exertion; in the night of the 8th of December, she became ill, having a severe pain in the region of the left knee, was feverish, and unable to walk or attend to her work. In a very short time a præpatellar inflammation developed, spreading rapidly upwards and downwards from the knee. On the 14th of December she was brought to the clinic; she already made the impression of being intensely septically infected; the inflammation spread rapidly to the foot and hip, no trace of an injury being observable. On the 15th of December two incisions were made (at the patella and on the outer aspect of the thigh) giving exit to clotted blood mixed with ichorous but not very offensive pus, containing large quantities of latent micro-coccos chains. The phlegmonous dermatitis had, on certain portions of its sharply defined borders, a clearly marked redness, indicating its erysipelatous character; immediately above the heel a blister had formed, filled with bloody serum, which was full of latent strepto-coccos. The patient died on the 16th of December. At the autopsy the joints and bones of the left extremity were found to be healthy; otherwise no evidence of local disease was found. Death was evidently caused by septicæmia without any farther localization. From the autopsy report, with the exception of the lower extremities, nothing pathological could be discerned. About the result of the microscopical investigations of the liver and kidneys we shall report later.

All these cases have this in common viz: that the inflammation occurred after mechanical injury; that, therefore, a primary excitation of the inflammatory process through micro-coccos vegetations could not be thought of; in all the cases there were found only latent strepto- and micro-coccos in the pus, varying in quantity. In two cases the pus had an ichorous appearance, but was odorless only in the one case, and in two cases the pus was entirely odorless.

The different abscesses required from six to fourteen days for their development. They were soon circumscribed, and in the last case, (Dolcher) only, we had to deal with an acutely progressive case, and only this one proved fatal. Ignoring the *generatio æquivoca*, it can only be assumed that coccos germs capable of vegetation were existing within the body, and that they developed in the site of the inflammation caused by an injury, probably because the pathological conditions especially favored their development at that point.

That the course of the disease was very mild in three of the cases mentioned, and exceptionally rapid in one of them, is somewhat difficult to understand.

The kind and degree of the injury, defective nursing, and improper treatment at the beginning of the disease, causes or conditions which usually serve to explain the difference in the result, cannot here be adduced. We are therefore left to the indefinable individual disposition to inflammations, if we do not wish to accept the modern but badly muddled theory of the different actions of the different varieties of micro-organisms, according to which man must carry within himself all kinds of micro-organisms in his tissues or blood, and when just those happen to vegetate that carry with them intensely inflammatory ferments, these would cause in the seat of irritation a serious progressive inflammation. This would compel us to assume again that it is not the vital vegetative act of the micro-organisms in question, that produces the ferment, but that there are only certain morphological not discernable organisms that do this, or to which the ferment in question is inseparably attached.

II. *Cases of coccobacteria vegetations in closed abscesses, in which there had existed a continuity with open wounds.* Under this heading belong several cases, which I have already communicated, of coccos in an abscess of the lymphatic glands of the neck after extirpation of the lip, etc. (Coccobacteria p. 89).

The last observation I made, was upon a boy æt. 14, June, 1874; also the following case belongs here. A man æt. 23, was operated upon for strangulated hernia. Three days after the operation the left half of the scrotum began to swell; the swelling increased rapidly, fluctuated distinctly, was completely isolated, could not be emptied even upon strong pressure until the original wound was punctured with a fine trocar. Five days after the first operation, it gave exit to about 8 grams of a yellowish clear odorless serum, and a few drops of pus. This fluid contained a few free medium-sized coccos and strepto-coccos, and was particularly infectious.

In all these cases the secretions were odorless, and there were always found only latent micro- and strepto-coccos; these organisms had gained entrance through the external wound, and from there entered the lymphatic circulation, and through it into the cellular tissue and lymphatic glands, and they carried with themselves infectious materials, and so aided the progress of the inflammation. We may, however, assume that an irritative lifeless ferment called forth the inflammation, and that the micro-organisms were accidentally developed just as in the primary sites of inflammation. This would be equally plausible.

The first hypothesis assumes a certain duration of the vegetative powers of these organisms in the living flowing lymph, which is verified by analagous experiences under different circumstances, and can therefore no longer be denied.

III. *Cases of coccobacteria vegetations in closed metastatic abcesses observed on the diseased.* New observations.

Carl Pash, æt. 23, injured himself four weeks before his reception (June 3, 1874), with a splinter of glass in the middle finger of the right hand; there was enormous inflammation and gangrene of the finger; the phlegmon extended up to the shoulder, suppurating already. The pus contained latent micro- and strepto-coccos in large quantities. On the 12th of July there occurred a swelling on the right natis, on a spot of which the patient had complained several days before; on the 13th evident fluctuation, a large quantity of foul smelling pus was drawn off that contained great quantities of free coccos and strepto-coccos; eight days later the pus which was discharged in small quantities contained only traces of coccos.

Haar Jacob, æt. 52. A medium sized hard calculus; urine, acid; slight cystitis; performed lithotripsy: first successful sitting on January 22d, 1876, no reaction. Second sitting January 24th; on the day following sudden severe pain during an attempt at micturition, severe hæmaturia, that persisted for a long time in spite of cold injections, and weakened the patient very much; (probably in consequenee of a plugging up by a sharp fragment) swelling of the corpora cavernosa, œdema of the prepuce, rapid development of an acute diphtheritic cystitis ; on the following day the urine, ammoniacal, containing many coccos elements; following this, œdema of the scrotum, partial gangrene of the same. Ammonemia on the 3d of February ; pain and then swelling of the right knee joint which was filled with pus, drawn off with the aspirator on the morning of the 8th of February. Exitus letalis on the evening of the same day. The extracted pus was thick yellowish and odorless, and contained great quantities of latent micro- and strepto-coccos.

Josef Kunz, æt. 6, received to be operated upon for eversion of the bladder and epispadias. Through repeated operations the bladder had been already closed, and the upper part of the penis and glans had become united. After the transposition of the prepuce upwards on the 23rd of June, the middle portion of the well-stitched prepuce became gangrenous; during the following days urinous diphtheria, alkaline bloody urine; on the 26th of June pain and swelling of the left knee joint. The joint was soon rendered tense by the accumulation of pus. On the 27th, puncture by means of the aspirator, gave exit to an odorless serous fluid, with a few flakes of pus, which contained great quantities of latent strepto-coccos coiled upon themselves. The fluid rapidly accumulated again; on the 5th of July, by means of puncture, thick, yellow, odorless pus, without micro-organisms was withdrawn, but the pus cells had a peculiar strongly marked granular appearance. Renewed accumulation ; punctured July 10th; no trace of coccos; the pus cells degenerating, accumulated again ; punctured with drainage July 24th. The pus was entirely free from coccos, and odorless; during the meantime the wound on the penis improved, becoming clean and granulating freely. The urinary diphtheria had passed away; urine, acid. During the time intervening between the 26th of June and the 5th of July, drops of blood drawn from the finger were repeatedly examined, but no clearly marked coccos, in fact not even a trace was found. The patient recovered; his joint was restored to normal mobility.

These investigations, it appears to me, offer much of interest. In all these four cases the metastatic disease could not well be be traced to blood clot, embolic from the primary inflammatory site containing coccos; therefore, it might seem questionable whether the vegetations in the metastatic abscesses were derived from those in the primary site of inflammation. We might assume that the coccos which are present in the pyæmically affected individual (as also in the healthy individual) readily vegetate, and that, therefore, there is no ætiological connection between the coccos in the wound and those in the metastatic abscess; that the coccos there as here is only an accidental addition to the disease itself. As important as they are to the poisoning of the secretions by acccidental or intentional inoculations, I incline to this view because of the cases already mentioned and others analogous.

If we desire to place the coccos in the primary abscess in direct (genetic) connection with the coccos in the metastatic abscess, then their transport was, even though it perhaps was begun by the tissue fluids, effected by means of the blood circulation. This would, as already indicated, imply a certain viability of the coccos in the circulating blood, but it would by no means prove that the eventual stopping of the coccos, for instance in the synovial membrane of the kneejoint, had caused the metastatic abscess. This might have originated from some other cause, simply offering an advantageous field for the development of the coccos as they were passing by.

Adopting either one of these hypotheses we come to another curious phenomenon. Were the coccos the carriers or the cause of the diphtheritic process in the two last mentioned cases (as has been assumed by many, and as I admit is possible from the experience obtained by its transmission to wounds after inoculations) then the passing cocci on their way to the knee joint lost their infectious diphtheritic character, because the pus in the knee joint was neither decomposing nor foul smelling; the process in the synovial membrane had a purely catarrhal blennorrhaghic character; there was no diphtheritic process; there followed in the last case *restitutio ad integrum.* I am not able to solve this enigma. As a positive, and as it appears to me, an important result of the observations, I call attention to the different results from the examination of the fluids extracted from the last case (Josef Kunz). In the, at first, serous secretions immense quantities of coccos were found, at the same time presenting no sign of decomposition; the more pus was formed the less coccos vegetations were found, at last disappearing altogether, the knee joint became normal again, the pus offered no peculiarity whatever to sight and smell.

That the coccos also disappeared in the ichorous pus, I have repeatedly called attention to. It appears to me that the cocci then decay when they have utilized the absorbed gases which the fluids contain; whether they have the power to decompose albuminoid bodies, still remains an hypothesis, notwithstanding assurances to the contrary, since the decomposition might occur without their presence.

The luxuriating coccos is therefore, when in the tissues of the body, confined to certain primary stages of the inflammatory process, they are destroyed by the rapid formation of pus. When, therefore, we find no coccos in ripened abscesses, we cannot say there never were any there. We can therefore not make any far reaching conclusions with regard to their action or their being the causative element in closed abscesses.

If these few experiments do not prove much, they at least agree completely with observations made during former experiments, that the secretions derived from the earlier period of an inflammation are phlogistically more irritating than those of later periods, and that the action of the coccos vegetations does not depend upon the mechanical influence, which they exert upon the tissues, nor upon the vital act of their vegetation, but upon this question, viz, whether or not they carry irritative materials with them into the tissues.

---

COMPOSITION OF PANCREATIC JUICE.—Defresne (*Rep. de Pharmacie*) has separated three different ferments from the pancreatic juice, each of which has different properties: *Amylopsine* converts starch into sugar; *Steapsine* splits up the fatty substances; *Myopsine* dissolves albumen.

